# Suppuration

**Published:** 1869-02

**Authors:** W. H. Thrift


### SUPPURATION.

BY W. H. THRIFT, D. D. S.

We constantly read articles on the different modes of treatment of different diseases, and seldom have the pathological facts of the case presented at all.

I shall endeavor, by this article, to give the reader as clear a pathology on suppuration as my limited knowledge will permit.

Suppuration is a result of inflammation, and is a work of destruction, and is, therefore, in a certain sense, to be contrasted with the effusion of lymph, which is intended to be a process of construction or reparation.

Suppuration is often useful, by terminating inflammation; it removes superfluous products and parts that have been injured by it, or its causes, and yet suppuration is an exhausting process.

In suppuration the inflamed vessels are more or less destroyed, while their supplying trunks are obstructed by lymph. In cases where the process of suppuration is limited by solid effusion, which is either the remains of the early products of inflammation, or it may be thrown out expressly for the purpose of defending the adjoining parts from the action of the pus. Pus thus formed is called an abscess.

The occurrence of suppuration is indicated by heat, pain, increased action, and other signs of irritation. The pain is often of a throbbing character, and more severe where surrounded by bony walls, as is the case in alveolar abscess. As suppuration advances, the swelling becomes softer, and if within reach so as to be felt by the finger, at first it will yield under the pressure, and afterwards will be felt the fluctuations of fluid matter. The redness present in inflammation is diminished in the part where there is suppuration; this is due to the suppurating mass. In external inflammations the redness of the skin becomes deeper before suppuration, and when suppuration reaches the skin, a pale spot is seen, which by its fluctuating feel, is an indication that it will soon make an external opening for exit.

Alveolar abscesses are, in most cases, due to a diseased condition of the tooth pulp, in which we may have a determination of blood to the pulp, and on account of the smallness of the foramen at the end of the root, congestion may follow, then inflammation and suppuration, which will terminate in the formation of an abscess. Dr. Taft, in his work on Operative Dentistry, says that the common cause of abscess is the presence of irritating matter in the canal at the point of the root, which no doubt in many cases extends through the foramen at that point, inducing inflammation and resulting in abscess.

I think I have shown that an abscess is the result of suppuration, and that suppuration is the result of inflammation.

I trust the foregoing remarks will prove suggestive, and assist in drawing attention from the profession to a subject of great interest.

